# Patch testing in occupational dermatology: Practical aspects in relation to the conditions in Germany 

**DOI:** 10.5414/ALX2483E

**Published:** 2024-05-03

**Authors:** Richard Brans, Christoph Skudlik

**Affiliations:** 1 Institute for Interdisciplinary Dermatologic Prevention and Rehabilitation (iDerm) at the Osnabrück University, and; 2 Institute for Health Research and Education, Department of Dermatology, Environmental Medicine and Health Theory, Osnabrück University, Osnabrück, Germany

**Keywords:** patch test, allergic contact dermatitis, delayed-type hypersensitivity, occupational dermatology, occupational skin disease, test quality

## Abstract

Allergic contact dermatitis is one of the most frequent occupational skin diseases. Targeted allergen avoidance can only be achieved by identification of the causative allergen. Therefore, patch testing is of utmost importance in occupational dermatology, not only in terms of assessing causal relationships but also regarding the implementation of prevention measures and evaluation of the legal criteria for an occupational skin disease in Germany (statutory occupational disease BK 5101). The lack of commercial patch test preparations poses a great diagnostic challenge. Patch testing of patient’s own materials from their workplace is therefore very important to reduce diagnostic gaps. The performance and documentation of the patch test should be in line with current guidelines and recommendations to ensure the necessary test quality and comprehensibility of the test results.

## Introduction 

Allergic contact dermatitis is one of the most common occupational skin diseases and is associated with severe skin lesions and a poor prognosis with regard to remaining in the profession [1, 2]. It is based on delayed-type hypersensitivity (syn. type IV hypersensitivity) reactions and is usually caused by low molecular weight substances and metal ions [3]. In addition to metals (e.g., nickel, cobalt, chromate), other important allergen groups include fragrances, preservatives/biocides, rubber ingredients or resins/adhesives [4]. The epicutaneous patch test is the diagnostic gold standard for the detection or exclusion of delayed-type hypersensitivity and thus allergic contact dermatitis. Only if the causative contact allergen is identified, re-occurrence of allergic contact dermatitis can be prevented through targeted allergen avoidance. The patch test is therefore of great importance in occupational dermatology, particularly with regard to the assessment of causal relationships between the skin disease and occupational activities as well as with regard to the implementation of preventive measures. In addition, the result of the patch test has an impact on the assessment of the severity of an occupational skin disease pursuant the German insurance law and thus the recognition of an occupational disease (Berufskrankheit: BK) BK 5101 of Annex 1 of the Occupational Disease Ordinance (Berufskrankheitenverordnung: BKV) as well as the assessment of the reduction in earning capacity (Minderung der Erwerbsfähigkeit: MdE) and the obligation to cease a harmful occupational activity. Even if the objective obligation to cease a harmful activity is no longer a prerequisite for the recognition of a BK 5101 since the reform of the German occupational disease law that came into force on January 1, 2021, the question of whether the continuation of the harmful occupational activity (even with recognized BK 5101) is possible from a medical point of view, or whether the accident insurance institution should urge the affected person to cease the harmful occupational activity (e.g., through retraining), continues to be of great importance with regard to preventive aspects. In many cases, the result of the patch test plays a key role in the assessment of the obligation to cease the harmful occupational activity and the resulting consequences. 

## Patch testing in occupational dermatology 

In occupational dermatology in Germany, diagnostic measures, in particular patch testing, can be carried out both in outpatient care, which is regulated in the so-called dermatologist’s procedure, and in legal evaluations as part of an expert opinion [5, 6, 7]. In the dermatologist’s procedure, workplace-related patch testing appropriate to the individual case can already be carried out at the expense of the accident insurance provider when the initial report (form F 6050) is submitted and thus before a treatment assignment is issued in accordance with § 43 of the contract pursuant to § 34 (3) of the German Social Security Code (SGB) VII between the National Association of Statutory Health Insurance Physicians and the accident insurance providers (https://www.kbv.de/html/uv.php). In view of the reform of the German occupational disease law that came into force on January 1, 2021, there are now different constellations in the dermatologist’s procedure, namely dermatologist’s procedures before recognition of a BK 5101, dermatologist’s procedures after recognition of a BK 5101 with continuation of the harmful occupational activity, and dermatologist’s procedures after recognition of a BK 5101 but without continuation of the harmful occupational activity [7]. Depending on the individual disease course, patch testing may be indicated regardless of the respective constellations. 

## Patch testing procedure 

The patch test should be performed in accordance with the evidence-based S3 guideline “Epicutaneous patch testing with contact allergens and drugs” (Reg. No. 013-018), published in March 2019, which was prepared under the leadership of the German Contact Dermatitis Research Group (DKG) and is available on the website of the AWMF (Association of the Scientific Medical Societies in Germany) (https://www.awmf.org/leitlinien/detail/ll/013-018.html) [8, 9]. Before carrying out the test, it is essential to take a detailed patient history in order to determine exposures to contact allergens that may be relevant at work or outside of work. For this purpose, it may also be necessary to review of the products the patient is exposed to (e.g., manufacturer information, safety data sheets). In patch testing, the so-called baseline series, which contains the most important allergens, should be tested in every patient. Based on the patient’s history and the possibly relevant contact allergens identified, further test series should be selected in which the test allergens are grouped thematically (e.g., occupation, allergen group) such as the DKG series for “hairdressing” or “construction industry”. The composition of the various test series is regularly updated by the DKG in cooperation with the Information Network of Departments of Dermatology (IVDK) (https://dkg.ivdk.org/testreihen.html). Testing should not exceed the necessary scope. Therefore, as a rule, with the exception of expert opinions, no more than 100 individual test preparations can be tested per case. In the online guide “Fees in occupational dermatology – A guide for billing from A to Z” (“Honorare in der Berufsdermatologie - Ein Leitfaden für die Abrechnung von A bis Z”) published by the German Social Accident Insurance (DGUV), there is a table that indicates which DKG series should be tested for important occupational groups (https://publikationen.dguv.de/widgets/pdf/download/article/3207). 

In contrast to the dermatologist‘s procedure, in which the dermatologist is authorized to perform the diagnostics exclusively to clarify the causal relationship between the skin disease and the occupational activity and thus the scope of the test is specified accordingly, the diagnostics in legal evaluations as part of expert opinions go beyond the work-related exposures: In order to exclude competing causes, it is appropriate in these evaluations to consider not only occupational but also non-occupational or private exposures when selecting the patch test preparations. Therefore, in contrast to the dermatologist‘s procedure, there is no limit to the number of patch test preparations which could be included in patch testing for expert opinions [5, 6, 10]. 

With regard to practical aspects of patch testing, the aforementioned patch testing guideline should be followed [8, 9]. Patch testing is usually performed on the patient’s back. It is recommended to leave the test chambers on the skin for 48 hours [11]. However, in individual cases (e.g., testing of irritant substances from the patient’s workplace) it may be advisable to limit exposure to 24 hours. The first reading is usually taken 15 – 30 minutes after removing the patch. A further reading on day 3 (72 hours) or day 4 (96 hours) is mandatory. If this is not done, the patch test is not viable. Due to few very late reactions, an additional reading between day 7 (168 hours) and day 10 (240 hours) is advisable. 

## Reading and interpretation of the patch test 

For the classification of test reactions, reference is made to the DKG reading criteria, which are based on morphological criteria [9]. The differentiation between (non-allergic) irritant, questionable, and only weakly positive (allergic) reactions can pose a particular challenge. This applies in particular to so-called problem allergens, which have an inherent potential for skin irritation and therefore often lead to false-positive reactions [12]. Testing of sodium lauryl sulfate (SLS) (0.25% aq.), which is also part of the DKG baseline series, is helpful in interpreting the results. If reddening occurs in the test area when testing this obligatory irritant, this indicates an increased irritability of the skin at the time of testing at the test site. In this case, weakly positive test reactions, especially to problem allergens, should be critically scrutinized and rather evaluated as presumably false-positive. A confirmation test (e.g., repeated open application test (ROAT)) may contribute to further clarification in individual cases [13]. 

## Relevance assessment and further procedures 

In the case of a positive test reaction evaluated as allergic, the relevance assessment is of great importance. Therefore, the relevance assessment of positive patch tests is not only mandatory in legal evaluations for BK 5101 as part of an expert opinion but is also explicitly required in both dermatologist report forms F 6050 and F 6052. For each detected delayed-type sensitization, it must be clarified whether it has a current or former occupational or non-occupational relevance in the present case and thus whether current or previous episodes of allergic contact dermatitis can be attributed to it. Not every positive test reaction is related to the skin lesions that led to the patch test. It is often necessary to take a new patient’s history or check additional of the products the patient is exposed to in order to assess the relevance. Unfortunately, safety data sheets are not always available online, are sometimes inaccurate in their information or do not always list all ingredients, especially if they fall below certain limit values. It is therefore often necessary to make specific inquiries to manufacturers to clarify whether a particular allergen is present in an occupational product. It should also be noted that some allergens are only mentioned with their synonym in the safety data sheet (e.g., 2-aminoethanol instead of monoethanolamine in metalworking fluids). If anything remains unclear, the responsible accident insurance provider should be advised to arrange for the so-called Prevention Service to carry out targeted research into the occurrence of the allergen at the workplace of the person(s) tested in order to assess the relevance of a positive patch test reaction. If the allergen is present in an occupational product, priority should be given to checking whether this can be replaced with another product that does not contain the relevant allergen (e.g., replacement of a metalworking fluid). This requires the cooperation of the employer. If a replacement is not possible, it must be checked whether skin protection measures for consistent allergen avoidance (e.g., adequate protective gloves) are available or can be implemented. Depending on the allergen and the type of allergic contact dermatitis (e.g., airborne), this can be a major challenge and, in individual cases, may render it necessary to cease the occupational activity if suitable preventive measures are not available. Evidence of an allergy to an unavoidable allergen in the workplace can therefore result in an objective obligation to cease a harmful activity [6]. In addition, it must be taken into account that since the reform of the German occupational disease law that came into force on January 1, 2021 and the concomitant abolition of the obligation to cease the harmful occupational activity as a prerequisite, the assessment of the severity of an occupational skin disease is of particular importance for the recognition of a BK 5101 and that an allergy to an unavoidable allergen at the workplace is an essential criterion for the new assessment of severity of an occupational skin disease pursuant to the BK 5101 [14]. 

## Remuneration for patch testing 

Remuneration for patch testing at the expense of the accident insurance providers in Germany is based on the currently agreed schedule of services and fees, the UV-GOÄ (Gesetzliche Unfallversicherung – Gebührenordnung für Ärzte). The UV-GOÄ contains significant differences to the fee schedule of private and statutory health insurance companies. With regard to the billing of patch tests, this means that the services within the framework of the UV-GOÄ are specified as individual tests and not as a number of points, according to which an amount is then shown in euros (standardized evaluation scale). When reimbursing patch testing in accordance with the UV-GOÄ, the testing of test substances outside the baseline series is also remunerated separately by means of surcharges. The UV-GOÄ distinguishes between a cost framework in the sense of general medical treatment and a cost framework in the sense of special medical treatment. In legal evaluations as part of an expert opinion, patch testing is generally billed according to the rates for special medical treatment; in the dermatologist’s procedure, it is generally billed according to the rates for general medical treatment [7, 10]. 

## Availability of commercial patch test preparations 

Allergen preparations for patch testing are diagnostic allergens (as opposed to therapeutic allergens) and are classified as medicinal products by EU-Directives 89/342/EEC and 2001/83 EC as transposed into national law in accordance with § 2 (1) of the German Medicinal Product Act (AMG). According to § 21 (1) AMG, a medicinal product may only be marketed in Germany if a national marketing authorization has been granted by the competent higher federal authority or if an authorization for all EU member states has been granted by the European Commission in accordance with Regulation (EC) No. 726/2004 [15]. In most cases, national marketing authorizations for diagnostic and therapeutic allergens are requested by the pharmaceutical companies. The competent higher federal authority in Germany is the Paul Ehrlich Institute (PEI). The German S3 guideline on patch testing recommends using pharmaceutically tested allergen preparations that are approved or marketable as medicinal products, if available [9]. It should be noted that since 2015 only one manufacturer has been selling approved/marketable patch test preparations in Germany. Since then, numerous authorizations have been withdrawn or have expired due to the so-called “sunset clause” (§ 31 para. 1 sentence 1 no. 1 AMG), as the test allergens have not been marketed as medicinal products for 3 years. In addition, market authorizations for new patch test preparations are hardly ever applied for. Manufacturers primarily refer to economic reasons for this, as the associated effort and costs are not profitable considering the expected turnover. As a result, the number of approved/marketable patch test preparations has decreased significantly in Germany in recent years. In addition, some approved/marketable patch test preparations are (temporarily) unavailable according to the manufacturer. Among other things, reference is made to changes in the raw materials supplied, which do not meet the specifications required in the market authorization of the test preparations. For example, the fragrance mix tested in the DKG baseline series has not been available in Germany for some time. According to the PEI, 205 patch test preparations had a valid market authorization in Germany on March 9, 2024 (compared to 343 patch test preparations in 2011). In addition, another 41 patch test preparations were in the ongoing authorization process under a transitional provision and were therefore marketable until the decision on authorization in accordance with § 141 (4) AMG (as of January 11, 2024). However, of these potentially available 246 patch test preparations, only 116 were available on March 9, 2024. This limited availability significantly compromises the informative value of patch tests. Only if the causative contact allergen is tested and thereby identified, re-occurrence of allergic contact eczema can be prevented through targeted allergen avoidance. The quality of the diagnostic work-up is therefore directly dependent on the availability of high-quality patch test preparations. 

## Patch testing of patient’s own workplace materials 

The range of available commercial patch test preparations is likely to remain limited in the long term. This will result in a considerable diagnostic gap in patch testing. It should also be noted that the allergen spectrum is large (> 5,200 described contact allergens) and allergen exposure at workplaces is subject to constant change [16]. New allergen exposures occur as a result of newly developed products or product adaptations, while others become less important, e.g., due to regulatory measures. 

In order to close the diagnostic gap, missing patch test preparations could be manufactured in pharmacies. According to § 13 (2) AMG, pharmacies do not require a manufacturing authorization for the production of medicinal products within the scope of normal pharmacy operations. However, this is associated with difficulties with regard to the procurement of raw materials, the associated costs, and the regulatory framework (e.g., pharmacy operating regulations). 

Another possibility to close diagnostic gaps is the supplementary patch testing of the patient‘s own workplace materials [17, 18]. According to § 13 para. 2b AMG, the individualized production of test preparations by the physician for a specific patient is possible. This allows the testing of the patient‘s own material (e.g., metalworking fluids), which formally becomes a medicinal product through testing. It should be noted that physicians who perform individualized patch tests with test preparations not approved as medicinal products (patient‘s own material) are required to once notify the respective competent state authority (GCP inspectorate) in accordance with § 67 (1) AMG [15, 19]. The DKG website provides a sample letter and information about which authority the letter should be sent to (https://dkg.ivdk.org/leitlinien.html). 

For patch testing patient‘s own material, prior commissioning by the accident insurance provider for the individual case is necessary. This requires prior notification of the number of planned tests and the patient‘s own material to be tested. The costs associated with the testing of the patient‘s own materials are remunerated separately in accordance with UV-GOÄ No. 379. An additional €5.60 or €11.20 can be charged for each material tested, depending on whether or not specific preparation is required for the production of the test substance. 

Special care and expertise are required when selecting and preparing the patient‘s own materials for patch testing. Some occupational substances (e.g., petrol, chalk, cement, solvents, rust removers) should not be patch tested due to their irritant/caustic effects or carcinogenic potential (Table 1). It should also be noted that substances or substance mixtures of unknown chemical identity or unknown biological effect or unknown, possibly excessive concentration should not be tested [9]. For many occupational substances, special preparation such as adequate dilution, selection of a suitable test vehicle, or control of the pH value is required for patch testing [20]. Assistance and instructions for patch testing patient’s own material can be found in the literature [16, 21, 22] and on the DKG website (“Instructions for patch testing with patient’s own material” (“Arbeitsanweisung Epikutantest mit patienteneigenem Material”); https://dkg.ivdk.org/leitlinien.html; last accessed: March 9, 2024). Recommendations for the preparation of important occupational materials and other patient’s own materials for patch testing are presented in Tables 2 and 3. 

Correct documentation of the substances tested and the test modalities is just as important as correct performance of the test. Only in this way can the quality of the testing and the viability of the test results be evaluated and appreciated. The DGUV‘s “Test sheet for patient’s own workplace materials” (“Testbogen Arbeitsstoffe”) shows what must be considered and documented when testing patient’s own workplace materials (https://www.dguv.de/medien/inhalt/versicherung/berufskrankheiten/hauterkrankungen/testbogen-arbeitsstoffe.pdf). The use of this test sheet is also a prerequisite for billing the testing of patient’s own materials in accordance with UV-GOÄ No. 379. The correct documentation is part of the service and therefore cannot be billed separately. 

As shown by the interim results of a currently conducted research project funded by the DGUV (FB 317b), there are considerable shortcomings in the quality of the documentation and performance of patch testing of patients’ own material in Germany [23]. In this project, data from 460 patients from all over Germany for whom test protocols of patch tests with patient‘s own material had been received by the accident insurance providers have been evaluated to date. In these patients, 3,004 individual tests with their own materials were carried out. The test protocols often lacked a full description of the material (26.7%) or, despite the requirement for diluting the material for patch testing, information on the test concentration (25.7%) or the test vehicle (29.5%) were missing. In addition, some products were tested that were not suitable for patch testing or that were not sufficiently diluted for patch testing. Patch tests carried out in medical practices were objected to more frequently than tests carried out in hospitals. Table 4 lists the most common avoidable mistakes in the performance and documentation of these kinds of tests. 

As most patient’s own workplace materials are mixtures of substances, it often remains unclear which single ingredient is the responsible allergen in case of a positive patch test reaction to the material. This can only be identified by patch testing of the relevant individual ingredients of the products. However, the range of commercial test preparations available in practices and clinics is often not sufficient for this so that the individual ingredients often have to be obtained for additional patch testing. This is hampered by insufficient manufacturer information on the ingredients, difficulties in obtaining them and the associated costs and time required. In addition, detailed research is often necessary to identify the optimal preparation of the individual ingredients for patch testing (e.g., test concentration, test vehicle). As a result, positive patch test reactions to mixtures are often not sufficiently clarified. This is unfortunate, as targeted allergen avoidance is only possible by identifying the causative allergen. 

## Conclusion 

In summary, patch testing is of great importance in occupational dermatology for the detection of delayed-type hypersensitivity. The limited availability of commercial patch test preparations and the increased efforts involved in testing patients’ own materials pose a particular challenge in the diagnostic work-up. To ensure high test quality and comprehensibility of the test results, care should be taken that the patch tests are carried out and documented in line with the current guidelines. 

## Authors’ contributions 

Both authors contributed equally to writing the original draft and to reviewing and editing the final version. 

## Funding 

No funding. 

## Conflict of interest 

The authors declare that there is no conflict of interest. 

**Table 1.** Examples of materials that should not be tested in a patch test.

Toluene	Antifreeze	Car wax
Gasoline	Diesel fuel	Kerosene/heating oil
Floor wax	Spirit	Solvent
Astringents (e.g. AgNO_3_)	Mineral acids (hydrochloric acid, sulphuric acid)	Alkali solutions
Chalk	Cement	Concrete

**Table 2.** Recommendations for the preparation of common occupational materials for the patch test.

**Substance**	**Concentration**	**Vehicle**	pH*	**Remark**
Hand and skin disinfectant	Pure (50%)	Water		
Surface disinfectant (use concentration)	0.1% 0.01%	Water		
Instrument disinfectant (use concentration)	0.01% (0.001%)	Water		
Metalworking fluid, water-soluble, used or unused concentration ≤ 8%	Pure (50%)	Water	X	24-hour occlusion Store sample in a cool place pH not > 9.5
Metalworking fluid, water-soluble, used or unused concentration > 8%	50% (10%)	Water	X	24-hour occlusion Store sample in a cool place pH not > 9.5
Metalworking fluid, water-soluble, concentrate	5%	Water	X	24-hour occlusion pH not > 9.5
Metalworking fluid, oily	50% (10%)	Olive oil		
Grease	Pure (20%)	Petrolatum		
Lubricating oil	50% 10%	Olive oil		
Gear oil	10%	Olive oil		
Hydraulic oil	(50%) 10%	Olive oil		
Silicone spray	10%	Petrolatum		
Wood dust	Pure	moistened		Exotic woods 1 : 10 petrolatum

*pH value must be checked before testing (pH value 5 – 8 is unproblematic).

**Table 3.** Recommendations for the preparation of other patient’s own materials for patch testing.

**Products**	**Concentration**	**Vehicle**	pH**	**Remark**
Moisturizer, emollient, sunscreens	Pure			Sunscreen: photo patch test may be required
Deodorants, perfume, hairspray	Pure			Drizzle/spray the product on a filter paper disk, let dry before application of test chamber
Hair gel	Pure			First semi-open test
Liquid soap, shampoo, shower gel	1% 5% (10%)	Water		
Shaving cream, gel, foam	1%	Water		
Household cleaners, detergents	0.1% 1%	Buffer*	X	
Clothing made of fabric, leather, rubber	Pure	moistened		

*Buffer solution pH 5.5 RN (DAB 10): Citric acid monohydrate 9.054 g, sodium monohydrogen phosphate 40.796 g, purified water ad 1,000 mL; **pH value must be checked before testing (pH value 5 – 8 is unproblematic).

**Table 4.** Frequent mistakes in the performance and documentation of patch testing patient’s own materials that severely limit the value and comprehensibility of the test results.

**Common mistakes**
Testing of products that are not suitable for patch testing
Full description of the product (name) is missing
Information on the test vehicle and/or the test concentration is missing despite dilution
Insufficient dilution / too high dilution
pH value is not measured or documented, even if required for testing (e.g., testing of metalworking fluids)
Reading times are not documented
Reading on day 3 (72 hours) or day 4 (96 hours) is not carried out or documented
